# Biomineralization-Inspired Synthesis of Cerium-Doped Carbonaceous Nanoparticles for Highly Hydroxyl Radical Scavenging Activity

**DOI:** 10.1186/s11671-017-2427-8

**Published:** 2018-03-06

**Authors:** Shenqiang Zou, Xiaofang Zhu, Lirong Zhang, Fan Guo, Miaomiao Zhang, Youwen Tan, Aihua Gong, Zhengzou Fang, Huixiang Ju, Chaoyang Wu, Fengyi Du

**Affiliations:** 1 Department of Hepatosis, The Third people’ s Hospital of Zhenjiang, Jiangsu University, 212021 Zhenjiang, People’s Republic of China; 2 School of Medicine, Jiangsu University, 212013 Zhenjiang, People’s Republic of China; 3 Department of Radiology, Affiliated Hospital of Jiangsu University, 212013 Zhenjiang, People’s Republic of China; 4 Department of Oncology, The Affiliated People’s Hospital, Jiangsu University, Zhenjiang, 212002 Jiangsu People’s Republic of China

**Keywords:** Cerium nanoparticles, Biomineralization, Hydroxyl radical scavenging

## Abstract

Cerium oxide nanoparticles recently have received extensive attention in biomedical applications due to their excellent anti-oxidation performance. In this study, a simple, mild, and green approach was developed to synthesize cerium-doped carbonaceous nanoparticles (Ce-doped CNPs) using bio-mineralization of bull serum albumin (BSA) as precursor. The resultant Ce-doped CNPs exhibited uniform and ultrasmall morphology with an average size of 14.7 nm. XPS and FTIR results revealed the presence of hydrophilic group on the surface of Ce-doped CNPs, which resulted in excellent dispersity in water. The CCK-8 assay demonstrated that Ce-doped CNPs possessed favorable biocompatibility and negligible cytotoxicity. Using H_2_O_2_-induced reactive oxygen species (ROS) as model, Ce-doped CNPs showed highly hydroxyl radical scavenging capability. Furthermore, flow cytometry and live-dead staining results indicated that Ce-doped CNPs protected cells from H_2_O_2_-induced damage in a dose-dependent effect, which provided a direct evidence for anti-oxidative performance. These findings suggest that Ce-doped CNPs as novel ROS scavengers may provide a potential therapeutic prospect in treating diseases associated with oxidative stress.

## Background

Continuous and deregulated inflammation is considered to be a common step of numerous pathological processes [1]. Oxidative stress has been found in many clinical diseases where ROS has much higher expression than elimination of antioxidant enzymes [2–4], which finally is defined as an imbalance between oxidative and anti-oxidative performances. The highly reactive ROS have a tendency to produce free radicals (including superoxide (O_2_^−^), HO_2ˋ_ and hydroxyl (_ˋ_OH)) which cause damage to macromolecules in the organism, such as proteins and nucleic acids, whereas overproduction of ROS makes a critical difference in sustained oxidative damage, leading to significant destructions of cellular structures and functions, such as inflammatory infiltrates [5–7], the damage of cell membranes and DNA [8, 9], and even increasing cell death. Thus, oxidative stress plays a key role in the pathogenesis of macro vascular diseases [10–12], cancer, and some other nervous system diseases [13]. Consequently, it is an important and urgent problem to control the concentration of reactive oxygen free radicals in living organisms and keep them in a suitable range, which is also basic to decrease series of oxidative abnormalities.

Recently, cerium oxide nanoparticles (CeO_2_ NPs) have attracted great attentions due to their unique redox properties that were extensively applied in energy, catalysis, and biomedical fields [14–16]. Especially, CeO_2_ nanoparticles exhibited huge potential as ideal antioxidant to treat ROS-related diseases in biomedical applications [17, 18]. The antioxidant activity of ceria nanoparticles derives from their ability of scavenging free radicals, such as superoxide (•O^2−^) and hydroxyl radicals (•OH), and maintains the antioxidant effect as an enzyme for a sustained period [19]. There is increasing evidence that CeO_2_ nanoparticles usually have a fluorite-type crystal structure, and cerium ions possess the ability of reversible conversion between tervalence (3+) and quadrivalence (4+) [20]. The switch of Ce^3+^ to Ce^4+^ oxidation states could eliminate O_2_^−^ via superoxide dismutase (SOD)-mimetics, and •OH via redox reactions, whereas the Ce^4+^ to Ce^3+^ switch remove H_2_O_2_ via catalase (CAT)-mimetics [21]. However, physical and chemical properties of CeO_2_ NPs may influence biological behavior, including its bio-distribution, pharmacokinetics, toxicity, dissolution, and elimination [22]. Given the hypothesis that it is well established on the superficial chemistry of Ce-doped CNPs, the biological utilization of Ce-doped CNPs as a therapeutic approach still need to be broadly explored [23, 24].

This study concentrates on the development of novel cerium-based nanoparticles (named as Ce-doped CNPs) with excellent biocompatibility using simple and green synthetic route and further explores their feasibility as antioxidant agent for biomedical applications. A series of methods was employed to characterize the physical and chemical properties of the Ce-doped CNPs. Encouraged by the favorable performances, we further verified the biocompatibility of the prepared Ce-doped CNPs and used them as antioxidant reagents to eliminate oxygen free radicals using H_2_O_2_ induced model. Finally, the antioxidant mechanism of Ce-doped CNPs was revealed from cell apoptosis pathway using the live-dead fluorescent staining and flow cytometry. This study will provide a novel and efficient ROS scavenger to alleviate oxidative stress damage under pathological conditions.

## Experimental Section

### Materials and Reagents

Bull serum albumin (BSA), fluorescein diacetate (FDA), and propidium iodide (PI) were purchased from Sigma (New York, NY, USA). Fetal bovine serum (FBS) and Dulbecco’s Minimum Essential Medium (DMEM) were procured from Invitrogen China Limited (Shanghai, People’s Republic of China). Ce (NO_3_)_3_·6H_2_O, methyl violet (MV), and ferrous sulfate heptahydrate (FeSO_4_^.^7H_2_O) were purchased from Aladdin Reagent Corporation (Shanghai, People’s Republic of China), and hydrogen peroxide (30%) were obtained from Sinopharm Chemical Reagent Co., Ltd. (Beijing, People’s Republic of China). All chemicals were of analytical reagents and were utilized without further purification. De-ionized water was used in the experiments.

### Synthesis of Cerium Oxide Nanoparticles

Ce-doped CNPs were prepared by applying a slight modification of album-based bio-mineralization method as described in the document [24]. The process of synthesis is illustrated as follows: 1.25 g BSA was dissolved in 50.0 mL of de-ionized water and constantly stirred to form the transparent solution. Then, the solution of metal precursor 300 mM Ce (NO_3_)_3_·6H_2_O were slowly added under vigorous stirring, respectively. Simultaneously, 2.0 M NaOH was dissolved in 50 mL de-ionized water, which was employed for adjusting pH value of the mixture. The solution including NaOH should be slowly added is worthy of being paid attention to. Ce-doped CNPs could be formed after stirring energetically at PH 12 at 55 °C. A sufficient reaction time for the formation of Ce-doped CNPs was about 8 h; the system was the compound of brown, cooling to the room temperature naturally. The additional suspension was filtered with a 0.22-μm membrane (poly-ether sulfone) to deplete those large scale agglomerations. The former solution was finally dialyzed against water for 3 days in a dialysis bag with a 14-kDa molecular weight cutoff. The collected dialysis solution was freeze-dried using a vacuum freeze dryer. The Ce-doped CNPs powders were ultimately obtained and saved for the further characterization.

### Instruments and Characterization of Cerium Oxide Nanoparticles

Morphologies of Ce-doped CNPs were examined by transmission electron microscopy (TEM) on a JEM-2100 microscope (JEOL, Tokyo, Japan) under an accelerating voltage of 200 kV. The size distribution was studied by the ImagingJ software (National Institutes of Health, Bethesda, MD, USA). The chemical structures of Ce-doped CNPs were analyzed using a Fourier transform infrared (FT-IR) spectrometer (Nicolet Nexus 470, GMI, and Ramsey, MN, USA). The elemental composition was determined by elemental analysis performed via an X-ray photoelectron spectroscopy (XPS). X-ray diffraction (XRD) analysis was performed on a Rigaku D/MAX-2000 diffractometer (Japan) operated at 40 kV and 100 mA, with a slit of 0.5° and a scanning speed of 7° min^−1^, equipped with a Cu Kα radiation source (λ = 0.15418 nm). The UV–Vis absorbance spectra were recorded using a UV-2550 UV–Vis Spectrophotometer (Shimadzu, Kyoto, Japan).

### UV-vis Photometric Experiments

Ce-doped CNP antioxidant performance and free radical scavenging activity were assessed by UV-vis photometric experiments. The solutions of MV were prepared in de-ionized water at a concentration of 3.0 × 10^−4^ M. And 0.15 mM FeSO_4_^.^7H_2_O was dissolved as an alternative. The suspended solution of Ce-doped CNPs was reserved to use at concentrations of 10 μM by dispersing in 0.1 M Tris-HCl buffer at pH 5.0. Appropriately, ultrasonic processing can enhance the solubleness of Ce-doped CNPs. The reaction solutions for photometric embraced 3.0 × 10^−5^ M MV, 0.15 mM FeSO_4_^.^7H_2_O, 1.0 M H_2_O_2_, 0.1 M Tris-HCl buffer (pH 5.0), and 0.17 mM Ce-doped CNPs to reach a required volume of 10 mL (mix solution of MV/FeSO_4_^.^7H_2_O/H_2_O_2_/CeO_2_) in final. The absorbance of the reaction solution could be measured after incubating for 5 min at the room temperature.

### Cytotoxicity of Cerium Oxide Nanoparticles

Cell viability of Ce-doped CNPs was evaluated on VSMC and 7721 cells by the CCK-8 assay (Dojindo, Kumamoto, Japan) based on the cleaved tetrazolium ring and the descent of water-soluble tetrazolium salt of the amount of formazan dye by dehydrogenases in living cells. Briefly, VSMC and 7721 cells (1.5 × 10 ^4^ cells per well) were seeded into 96-well plates with five replicates for each group. After hatching for 24 h at 37°C and 5% CO_2_ and cell density had reached 80% confluence, the growth medium was replaced with fresh DMEM containing different concentrations (0, 12.5, 25, 50, 100, 200, 400 μg/ml) of Ce-doped CNPs solution and incubated for 24 h. Then, the cells were washed with PBS and 10 μL CCK-8 solution was added to each well. Next, the 96-well plates were incubated for another 4 h at 37°C and 5% CO_2_ to allow exponential cell growth. Finally, the absorbance of each well was detected at emission wavelength of 490 nm using Synergy HT Multi-Mode Microplate Reader (Bio-Tek, Winooski, VT, USA). Cells (in DMEM) without treatment were used as control, and the relative cell viability (mean ± standard error of mean) was calculated using Abs sample/Abs control × 100%.

### Model of Hydrogen Peroxide on Cells Viability Assay In Vitro

As a kind of potent oxidative active oxygen, hydrogen peroxide (H_2_O_2_) is easy to go through the cell membrane and reacts with intracellular iron ions to form highly reactive free radicals by Fenton theory, leading to a chain of changes. At the same time, varieties of cell oxidative stress induced by H_2_O_2_ are directly involved in the process of apoptosis. Therefore, 30% H_2_O_2_ was chosen to simulate the apoptosis of cells in this study, which also can be used to be an IC_50_ model to further investigate the protective effect of Ce-doped CNPs on oxidative damage. Based on the above discussion, VSMC and 7721 cells were initially seeded at a density of 1.5 × 10^4^ cells/well into the 96-well plates. After cells were adherent, they continued to be cultured for extra 24 h. And the fresh preparation 30% H_2_O_2_ was added to each well with different concentrations (50, 100, 200, 400, 800 umol/ml) to stimulate growth of cells. After a 4-h hatch and a subsequent wash with PBS, 10 μL CCK-8 solution was added to each well for 4 h at 37 °C in a 5% CO_2_ incubator_._ Synergy HT Multi-Mode Microplate Reader (Bio-Tek, Winooski, VT, USA) was employed to determine the absorbent value of each well at 490 nm. The percentage of cells activity was regarded as the index of the effect of on cell viability, which depended on measuring the cell survival rate relative to that of the control.

### Apoptosis Induction and Cell Viability Assay In Vitro

In the same way, VSMC and 7721 cells were seeded in the 96-well plates and cultured. The next day, cells were pretreated with different concentrations of Ce-doped CNPs (0, 12.5, 25, 50, 100, 200, and 400 μg/ml) about 24 h and exposed to the fresh preparation 30% H_2_O_2_ with the suitable concentration each well. On the other hand, it should be set a blank group without 30% H_2_O_2_ and Ce-doped CNPs and control group was only with H_2_O_2_ solutions. Each group was set up six parallels. After treatment for 4 h and a subsequent wash with PBS, the CCK-8 assay was used to measure the apoptotic conditions of cells each well at an optical density of 490 nm as the described above.

### Live-Dead Staining Assay and Flow Cytometry

To assess the potential of antioxidant effect of Ce-doped CNPs, 7721 cells were grown on six-well plates with 4.0 × 10^4^ cells in 100 ul media per well and allowed to develop for 24 h. Then, six groups were set to be simultaneous contrast; it included the control, H_2_O_2_ group, 50μg/mL Ce-doped CNPs + H_2_O_2_, 100μg/mL Ce-doped CNPs + H_2_O_2_, 200μg/mL Ce-doped CNPs + H_2_O_2_, and 400μg/mL Ce-doped CNPs + H_2_O_2_. While cells had grown to 80% in each group, the first group was exposed to a normal growth condition without H_2_O_2_ and Ce-doped CNPs; the second group was just incubated with the selected H_2_O_2_ without Ce-doped CNPs for 4 h, namely no treatment; the third group to the sixth group contained different concentrations of Ce-doped CNPs (50, 100, 200, and 400μg/mL) and indicated H_2_O_2_ incubating for 4 h, respectively. After that, FDA and PI working buffer was used for cell staining (Calcein AM/Ethidium Homodimer, Invitrogen). The fluorescence of stained cells was observed under a fluorescence microscope; live cells showed green color, and dead ones is red color.

Furthermore, the Annexin V-FITC/propidium iodide (PI) Apoptosis kit was used to quantify the rate of cell apoptosis by double fluorescence staining. Briefly, 7721 cells were pre-cultured in six-well culture plates according to the above steps for 24 h, until the 7721 cells adherent growth. After that, the cells with different doses of treatment measures were harvested and washed three times with PBS at 4 °C in each group. It was noticed that the cells should be gently digested, collected, and centrifuged followed by 2000 rpm for 5 min. Thereafter, the cells were resuspended with 500 μl binding buffer and adjusted to a concentration of 1 × 10^6^ cells/ml. And then, the cell suspension was taken into a flow tube staining with 5 μl of Annexin V-FITC and 5 μl of 20 μg/ml of PI solution, respectively. Before the mixture incubating at room temperature for 15 min, 500 μl PBS should to be added to the reaction tube. At last, the experimental results were detected using Annexin-V method on a Beckman Coulter Epics XL MCL flow cytometer system (BD Accuri C6), and the percentage of apoptotic cells was clarified by software analysis (Flowjo 7.6.2).

### Statistical Analysis

All the experimental data were expressed as the mean ± standard error of mean. For comparison of the different groups, statistical technique of ANOVA with the post hoc least significant difference (LSD) test was applied to analyze data that were obtained. A *P* value of less than 0.05 (*P* < 0.05) was considered to be statistically significant.

## Results and Discussion

### Preparation and Characterization of Cerium Oxide Nanoparticles

**Fig. 1:**
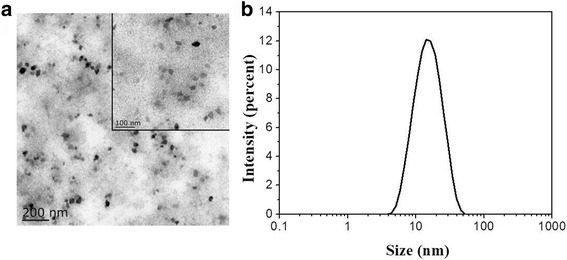
**a** TEM images for Ce-doped CNPs. The inset showed high-resolution TEM image. **b** The diameter distribution of Ce-doped CNPs

In this study, the aqueous dispersions of Ce-doped CNPs were obtained using Ce (NO_3_)_3_^.^6H_2_O and BSA as precursors by biomineralization-inspired synthetic method. During the preparation, BSA used as major carbon source materials can integrate the metal ions to metal nanoparticles due to their unique spatial structure and molecular chain flexibility. In other words, the prepared metal-doped nanoparticles are mainly composed of carbonaceous framework and loaded metal element, which may be different from conventional inorganic cerium nanoparticles [25]. The morphological examination was characterized by transmission electron microscopy (TEM), and the resultant particle sizes were analyzed by the ImagingJ software. Fig. 1a showed characteristic TEM images of the resultant Ce-doped CNPs. These imagines reflected that Ce-doped CNPs possessed polygonal structure, uniform dispersion, and discrete shape without apparent aggregation. Statistical analysis indicated Ce-doped CNPs had a narrow size distribution with an average diameter of 14.7 ± 0.8 nm (Fig. 1b), which was consistent with the TEM images. When Ce-doped CNPs were in water for a few days, it can be found that there was no precipitation and still kept the statement of homogeneous solutions, indicating their long-term stability in aqueous solution, which could be beneficial to the following biomedical applications.

**Scheme 1:**
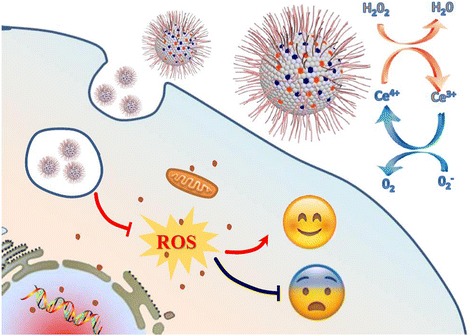
Schematic illustration of the design of Ce-doped CNPs

### The Chemical Structure and Surface Composition of the Ce-Doped CNPs

**Fig. 2:**
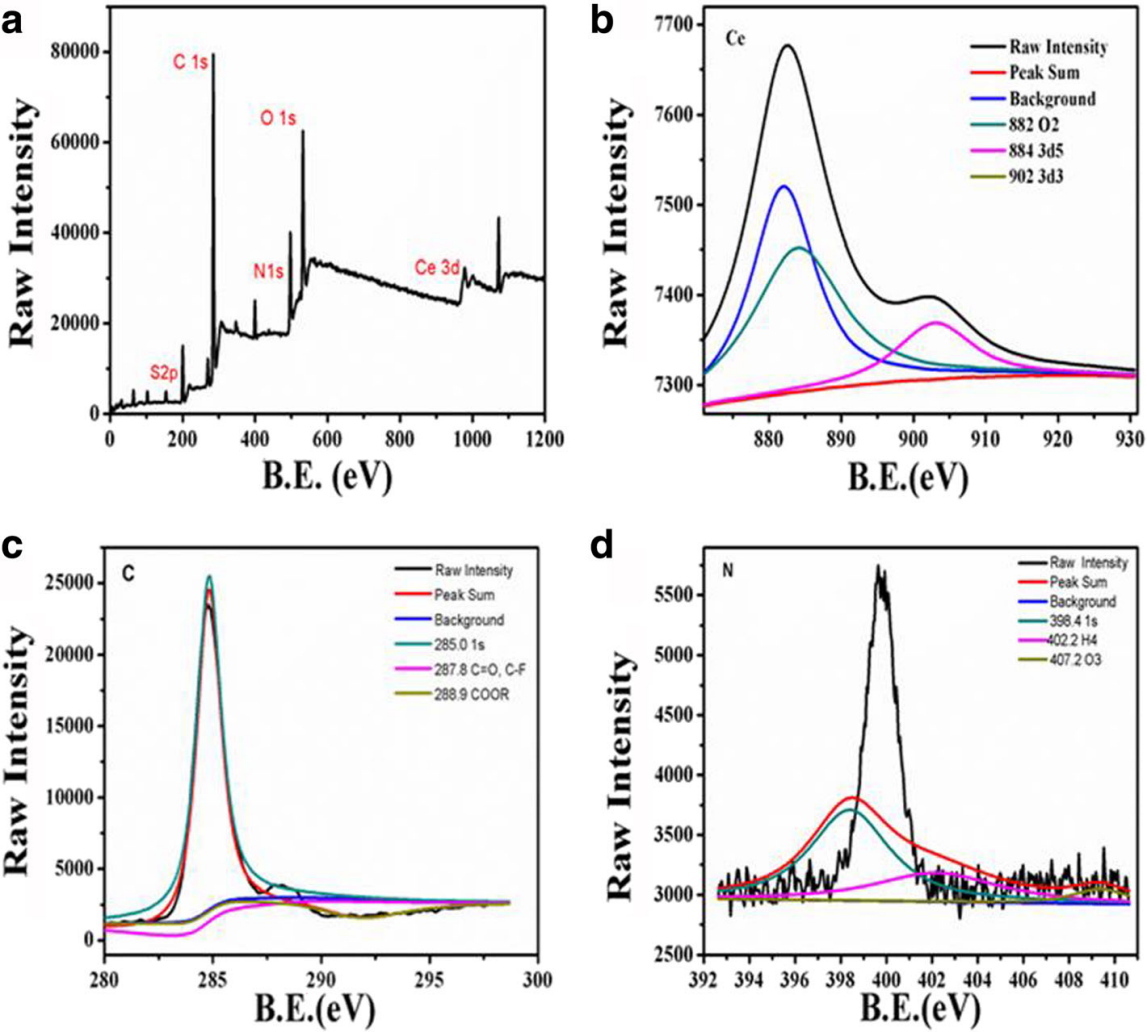
**a** XPS spectra of the Ce-doped CNPs. Survey spectrum. **b** Ce3d spectrum. **c** C1s spectrum. **d** O1s spectrum

Surface functional groups and composition of the Ce-doped CNPs were investigated using X-ray photoelectron spectroscopy (XPS) pattern and Fourier transform infrared (FT-IR) spectrum. XPS was used to characterize the oxidation state of cerium ions on the surface of samples. The survey XPS spectrum (Fig. 2a) showed three predominant peaks at 902.0, 285.0, and 531.8 eV, which indicated the existence of cerium, carbon, and oxygen elements of Ce-doped CNPs. The sulfur(S 2p) signals at 164.0 eV suggested that BSA was successful conjugated to the Ce-doped CNPs. The coexistence of Ce(III) and Ce(IV) in Ce-doped CNPs catalyst could be evidenced by the spectrum of the Fig. 2b. The high-resolution spectrum of Ce3d (Fig. 2b) could split into Ce3d_3_ and Ce3d_5_ (resulting from a spin–orbit splitting of 32 eV), exhibited the presence of indexed and strong peaks, located at 882.0, 884.0, and 902.0 eV, respectively. Ce3d signals were mainly divided into O_2_, 3d_5/2_, and 3d_3/2_, the peaks between 875 and 895 eV belonged to the Ce3d_3/2_ level, which almost coincided with the spectrum of previously published [26]. Moreover, the XPS spectrum also implied the 15.99% of Ce3d. These observations are in agreement with results in the literature for CeO_2_ nanoparticles [25]. The C1s spectrum as shown in Fig. 2c was described by three major peaks at 285.0, 287.8, and 288.9 eV, which respectively implied that CeO_2_ nanoparticles were functionalized with the 1s, C=O, C−F, and COOR energy levels of C. The presence of the O1s spectrum as shown in Fig. 2d was dominated by two major peaks at 530.5 and 531.8 eV, which corresponded to the bond between O_2_^−^ and Ce^3+^.

**Fig. 3:**
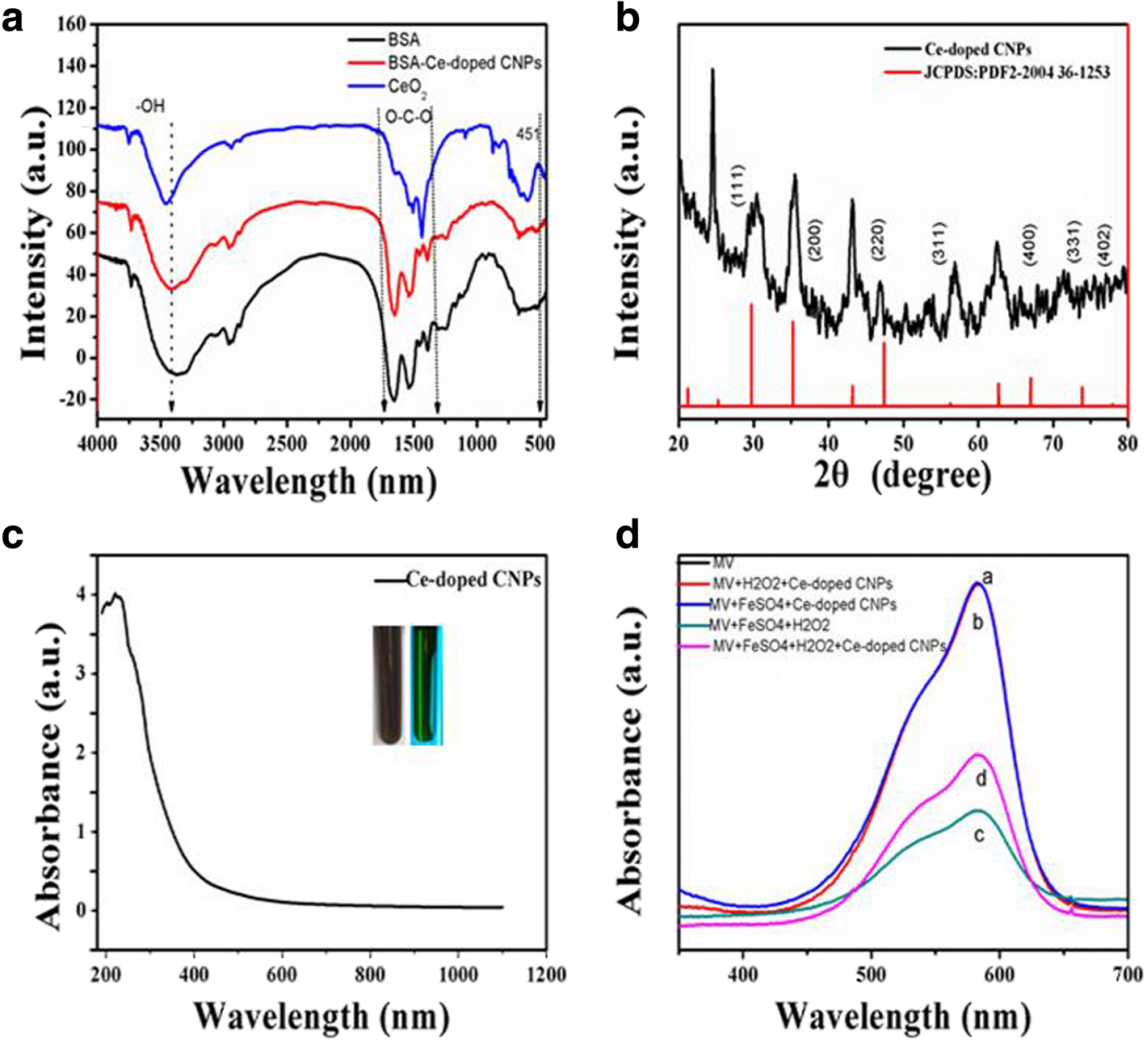
A FTIR spectra of BSA and Ce-doped CNPs. B XRD pattern of the Ce-doped CNPs. C UV–Vis absorption spectra of Ce-doped CNPs. D UV–Vis absorption spectra of Methyl violet (MV) after various treatment. **a** MV, **b** MV treated with H_2_O_2_ and Ce-doped CNPs, **c** MV treated with FeSO_4_ and H_2_O_2_, and **d** MV treated with FeSO_4_, H_2_O_2_, and Ce-doped CNP solutions at an incubation time of 5 min

FTIR analysis was further carried out to verify the formation of Ce-doped CNPs in the reaction process. As shown in Fig. 3A, for the Ce-doped CNPs, an intense and broad peak was appeared at 3400 cm^−1^, which was caused by the stretching vibration of O–H and could be assigned to the bending vibration of water absorbed. The characteristic peak at 1510 cm^−1^ could be described to the –O–C–O antisymmetric stretch, while the peak at 1450 cm^−1^ could be assigned to the symmetric stretching vibration of –O–C–O, both of which demonstrated the presence of nitrate groups. In addition, an absorption band in 451 cm^−1^ was assigned to the Ce–O asymmetric stretch, indicating the formation of Ce-doped CNPs. These results showed that the functional groups of Ce-doped CNPs mainly contained certain −OH and –COO^−^ groups.

According to X-ray diffraction (XRD) analysis, the crystal structure of the Ce-doped CNPs was investigated. As shown in Fig. 3B, there are four major diffraction peaks at 24.4°, 30.3°, 35.23°, and 43.2°. One of these peaks at 24.4° may be ascribed to the produced inorganic carbonaceous structure, which is similar to the reported carbon quantum dots and graphite characteristic peaks [27–29]. Compared to the ordered crystal structure of graphite (002 planes, 2*θ* = 26.5°), the diffraction peak of Ce-doped CNPs around 24.4° with a lower shift becomes weak, which may be attributed to highly disorder carbon and increase in the sp^2^ (C–C) layer spacing in the carbonization process [30]. The diffraction peaks at angles of 30.3°, 35.23°, and 47.42° mostly matched with (111), (200), and (220) planes, respectively, of the conventional ceria nanoparticles. Furthermore, other peaks at angles of 56.30°, 69.00°, 75.57°, and 78.99° were corresponding to (311), (400), (331), and (402) of referenced cerium oxide (JCPDS 2004 36-1253). The corresponding spacing $\left(\mathrm{Fm}3\overline{\mathrm{m}}\right)$ was calculated according to the Bragg’s law (the wavelength of Cu-Kα law is 0.154 nm). These results could preliminarily confirm the Ce-doped CNPs with the hybrid crystal structure.

It was well known that Fe^2+^ can alternately catalyze dismutation of the hydrogen peroxide (H_2_O_2_) into other reactive hydroxyl radical according to Fenton reaction [31]. As a chromogenic reagent, the solution of methyl violet mainly shows purple. Targeted with –C=C–, hydroxyl radical can react with methyl violet, resulting in MV into a shallow color even colorless, and its maximum UV-Vis absorbance at 582 nm decreases [32]. According to our design, Ce-doped CNPs certainly can eliminate the hydroxyl radical, finally increase the absorbance of the MV at 582 nm.

To confirm the underlying free radical scavenging capability for Ce-doped CNPs, UV-Vis absorption spectra of MV was employed to provide some insights. There was no significantly absorbance from 500 to 700 nm in Ce-doped CNPs aqueous solution in Fig. 3C. However, we could observe the maximum absorbance of the methyl violet at about 582 nm and the absorbance was inhibited under H_2_O_2_ treatment (Fig. 3D). When adding Ce-doped CNPs into mix solution, the absorbance was obviously recovered, which proved that Ce-doped CNPs had the ability to clear the hydroxyl radical and protect the MV against being attacked at this experimental condition. These results will have further biomedical application in treating diseases caused by free radicals.

### Cytotoxicity Assay of Cerium Oxide Nanoparticles In Vitro

**Fig. 4:**
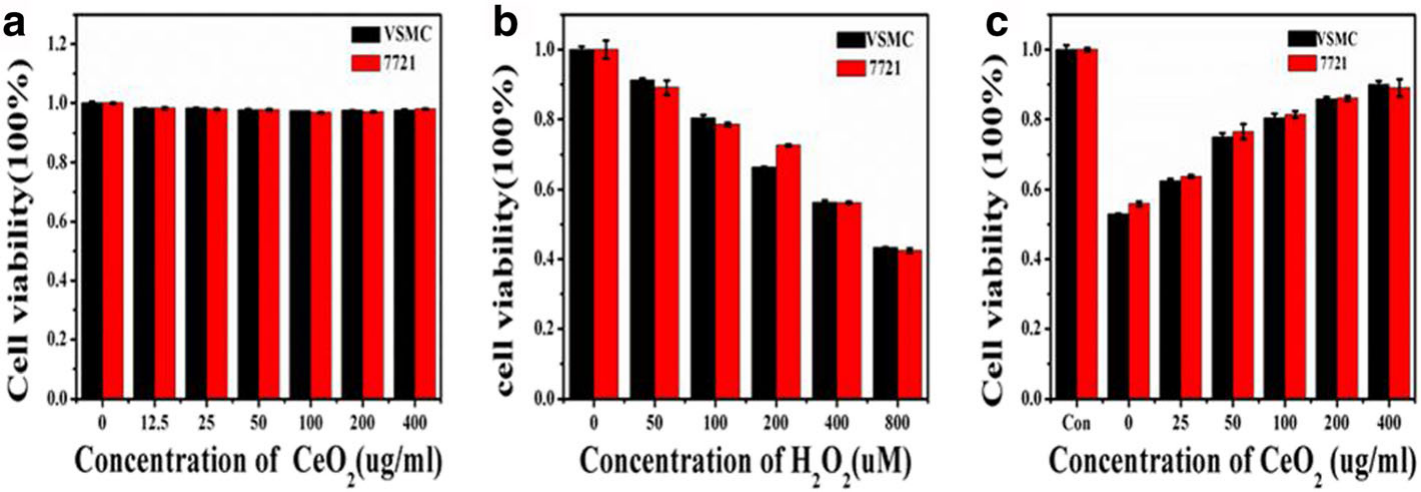
**a** The effect of various concentrations of Ce-doped CNPs on the cell viability of VSMC and 7721 cells by CCK-8. Cells were pre-treated Ce-doped CNPs at different concentrations for 24 h and then were administrated by H_2_O_2_ at different dosages. **b** Cell viability of VSMC and **c** 7721 cells cultured with different concentrations of Ce-doped CNPs under 560 μM H_2_O_2_-induced oxidative stress a by CCK-8 assay

Before applying synthesized Ce-doped CNPs to treat diseases associated with oxidative stress, it is essential to evaluate their biocompatibility. In this study, the effect of Ce-doped CNPs on cell viability were evaluated via incubation of VSMC and 7721 cells with Ce-doped CNPs using cell viability tests of the CCK-8 assay. It can be seen that the cell viability did not show significant changes after different concentration of Ce-doped CNPs treatment for a 24-h exposure time (Fig. 4a). Even at the highest dose of 400 μg/ml, the cell viability in all treatment groups was approximately 90%, demonstrating that Ce-doped CNPs had a very slight cytotoxicity on cells. These findings highlighted the favorable cyto-compatibility of Ce-doped CNPs and enabled them suitable for biomedical applications.

### Antioxidant Properties and Free Radical Scavenging Activity of Ce-Doped CNPs In Vitro

The property of mimetic enzyme of cerium oxide nanomaterials is conducive to their potential therapeutic applications. Whether Ce-doped CNPs in solution would sufficiently protect VSMC and 7721 cells against free radical was examined. For these studies, H_2_O_2_ was used to induce the reactive oxygen species (ROS) generation, which lead to cells impairment or death. Firstly, the IC_50_ value of cell viability was determined to explore the protective effect of Ce-doped CNPs. VSMC and 7721 cells viability exhibited the low value after the addition of H_2_O_2_. A trend of increased apoptotic activity for VSMC and 7721 cells was observed with increasing H_2_O_2_ concentration in solution (Fig. 4b). In other words, the elevation of ROS was dose-dependent, which also revealed the median lethal dose (LD_50_) of VSMC and 7721 cells was approximately 549 and 556 umol/ml, respectively. As a result, the LD_50_ H_2_O_2_ via oxidative stress was 560 μM, which was close to the results of the literature [33].

Next, the antioxidant effect of Ce-doped CNPs under LD_50_ of H_2_O_2_ was tested using a cell counting kit-6 (CCK-8) assay. Owing to their unique electronic properties, Ce-doped CNPs have an inherent tendency to exist in dual oxidation states (Ce^3+^ and Ce^4+^) and are conferred antioxidant activity via oxidation-reduction behavior. When ROS exists, Ce^4+^ can be continuously switched to Ce^3+^ along with the persistent free radical scavenging activity [34, 35]. As shown in Fig. 4c, these results implied that the cell viability of both VSMC and 7721 cells had been gradually improved with the increase of Ce-doped CNPs concentration under same oxidative stress. Especially, the cell viability was recovered about 88.0% at the Ce-doped CNP concentration of 400 μg/ml. Consequently, the Ce-doped CNPs exhibited an outstanding antioxidative effect in a dose-dependent manner via efficient free radical scavenging capability.

**Fig. 5:**
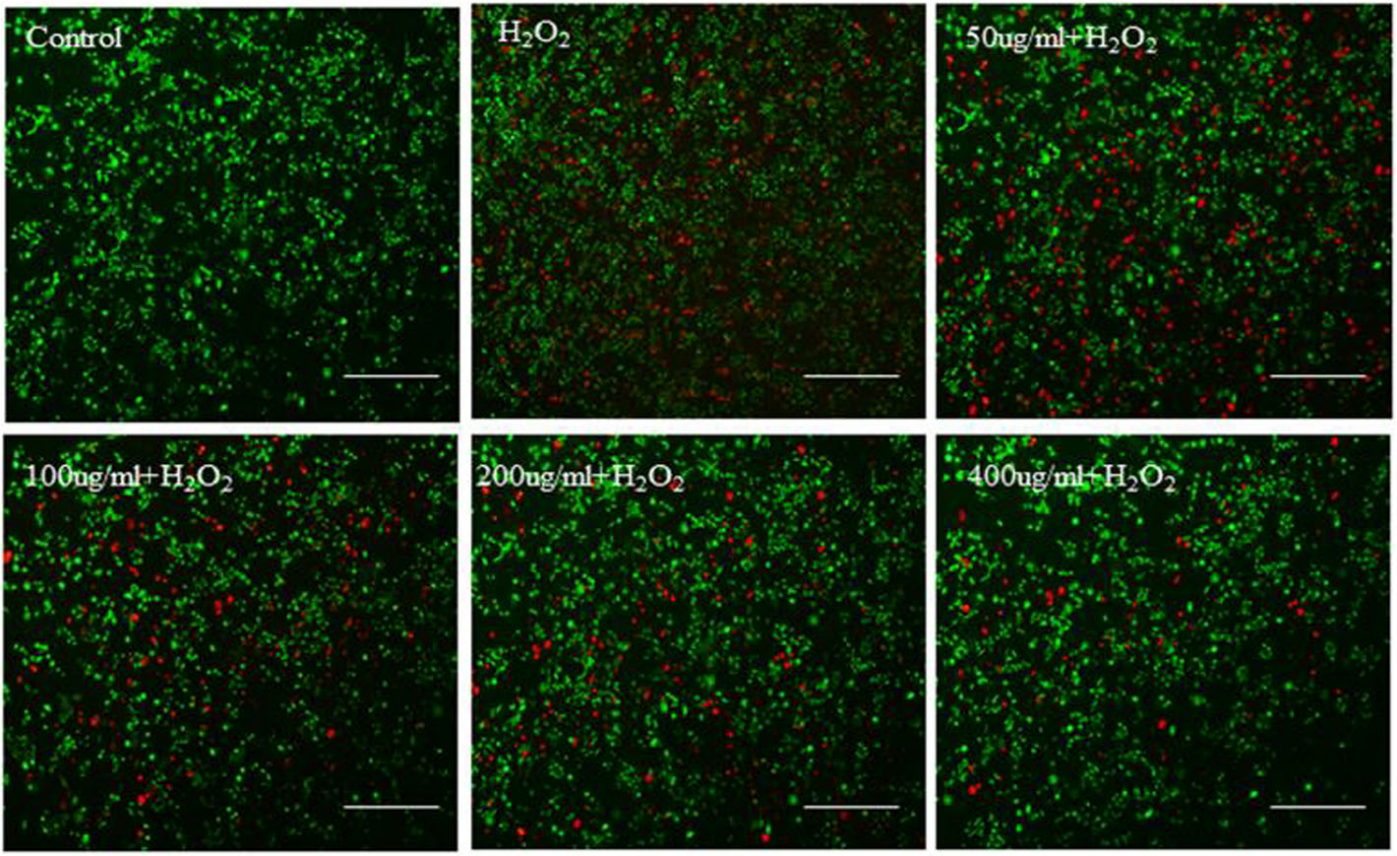
Fluorescent images of 7721 cells incubated with different concentrations of Ce-doped CNPs under H_2_O_2_-induced oxidative stress for 24 h by live-dead staining (scale bars = 50 μm)

**Fig. 6:**
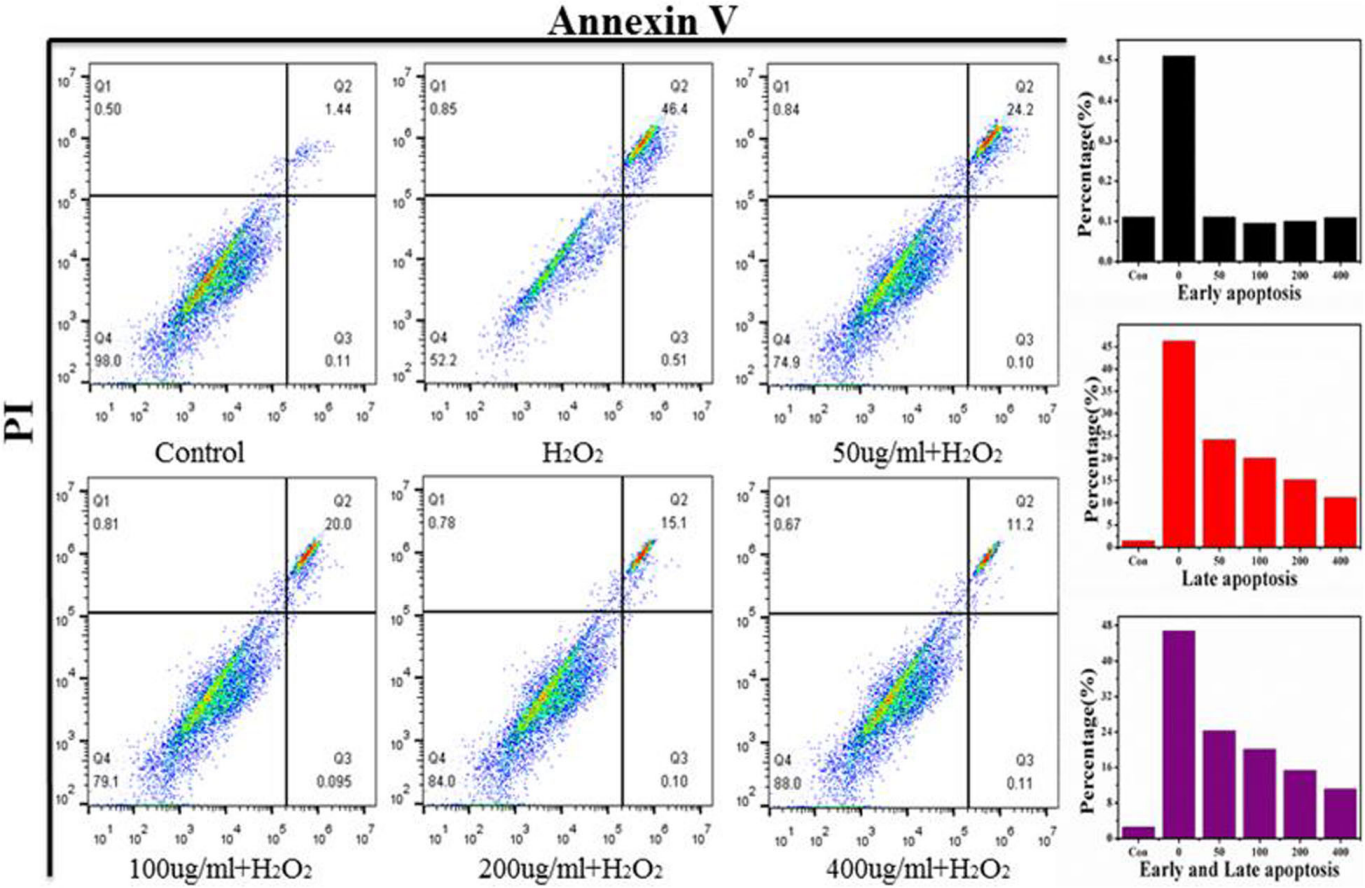
Flow cytometry profiles of 7721 cells were examined to determine the percentages of early apoptosis and late apoptosis cells with different treatments

Live-dead staining and flow cytometry assay was employed to further uncover the cyto-antioxidative effect of Ce-doped CNPs. As depicted in Fig. 5, intensive green fluorescence without red was observed in the 7721 cells treated with single Ce-doped CNPs, but a lot of red fluorescence could be seen in the H_2_O_2_ treatment of 556 uM, which demonstrated H_2_O_2_ could induce dramatically cell apoptosis. But H_2_O_2_-induced apoptosis had been gradually alleviated with the increase of Ce-doped CNP concentrations. In particular, only a small amount of red fluorescence could be seen after Ce-doped CNP treatment of 400 μg/ml under H_2_O_2_-induced oxidative stress which indicated higher cell viability. Flow cytometry assay was further carried out to investigate the antioxidative effect of Ce-doped CNPs. In the scatter plot of double variable flow cytometry, Annexin-V-FITC emission signal was plotted on the *x*-axis, while PI emission signal was plotted on the *y*-axis. Several regions of cells were defined as follows: Annexin V-/PI––are considered living cells, Annexin V+/PI––stands for early apoptosis cells, and Annexin V+/PI+––represents late apoptosis/necrosis cells. As displayed in Fig. 6, the control group exhibited a small and normal fraction of cell late apoptosis of 1.45%, but H_2_O_2_ treatment displayed an extremely high rate of cell late apoptosis of 46.4%. As we expected, the proportion of apoptotic cell treated by different concentration of Ce-doped CNPs has a gradual downward trend. Compared to 46.4% cell late apoptosis under the H_2_O_2_ treatment, the percentage of apoptosis markedly decreased to 24.2, 20.0, 15.1, and 11.2% after Ce-doped CNP treatment of 50, 100, 200, and 400 μg/ml. Basing on the results of the live-dead staining and flow cytometry, it revealed that Ce-doped CNPs exerted favorable antioxidative effects against oxidative stress-induced apoptosis in a dose-dependent manner.

## Conclusions

In summary, we reported a novel Ce-doped CNPs with the capability of scavenging free radicals. The preparation method is simple by using one-step synthesis in the bio-mineralization manner. Different from the former surface modification (such as PVP), the as-prepared Ce-doped CNPs exhibited the improvement in biocompatibility and dispersibility in aqueous solution. Furthermore, in vitro studies showed albumin-based bio-mineralization Ce-doped CNPs not only own superoxide dismutase feature but also alleviate the oxidative damage caused by H_2_O_2_, which have a protective effect on cells in this periods of study. Furthermore, the concentration of Ce-doped CNPs plays important roles in the recovery of apoptotic cells. Herein, the novel Ce-doped CNPs have promising biomedical applications in diseases prevention and treatment of oxidative stress-mediated.
